# The neurophysiological Behavioral Perspective Model of consumer choice and its contribution to the intentional behaviorist research programme

**DOI:** 10.3389/fnhum.2023.1190108

**Published:** 2023-08-01

**Authors:** Gordon R. Foxall

**Affiliations:** ^1^Cardiff Business School, Cardiff University, Cardiff, United Kingdom; ^2^School of Business Administration, Reykjavík University, Reykjavik, Iceland

**Keywords:** Behavioral Perspective Model (BPM), consumer choice, neurophysiology, dual process, valuation

## Abstract

Cognitive explanations raise epistemological problems not faced by accounts confined to observable variables. Many explanatory components of cognitive models are unobservable: beliefs, attitudes, and intentions, for instance, must be made empirically available to the researcher in the form of measures of observable behavior from which the latent variables are inferred. The explanatory variables are abstract and theoretical and rely, if they are to enter investigations and explanations, on reasoned agreement on how they can be captured by proxy variables derived from what people say and how they behave. Psychometrics must be founded upon a firm, intersubjective agreement among researchers and users of research on the relationship of behavioral measures to the intentional constructs to which they point and the latent variables they seek to operationalize. Only if these considerations are adequately addressed can we arrive at consistent interpretations of the data. This problem provides the substance of the intentional behaviorist research programme which seeks to provide a rationale for the cognitive explanation. Within this programme, two versions of the Behavioral Perspective Model (BPM), an extensional portrayal of socioeconomic behavior and a corresponding intentional approach, address the task of identifying where intentional explanation becomes necessary and the form it should take. This study explores a third version, based on neurophysiological substrates of consumer choice as a contributor to this task. The nature of “value” is closely related to the rationale for a neurophysiological model of consumer choice. The variables involved are operationally specified and measured with high intersubjective agreement. The intentional model (BPM-I), depicting consumer action in terms of mental processes such as perception, deliberation, and choice, extends the purview of the BPM to new situations and areas of explanation.

## Intentional behaviorism

Cognitive explanations raise epistemological problems not faced by accounts confined to observable variables. Many explanatory components of cognitive models are unobservable: beliefs, attitudes, and intentions, for instance, must be made empirically available to the researcher in the form of measures of observable behavior from which the latent variables are inferred. The explanatory variables are abstract and theoretical and rely, if they are to enter investigations and explanations, on reasoned agreement on how they can be captured by proxy variables derived from what people say and how they behave. Psychometrics must be founded upon a firm, intersubjective agreement among researchers and users of research on the relationship of behavioral measures to the intentional constructs to which they point and the latent variables they seek to operationalize. Only if these considerations are adequately addressed can we arrive at consistent interpretations of the data. This problem provides the substance of the intentional behaviorist research programme (Foxall, 2020) which seeks to provide a rationale for the cognitive explanation. Within this programme, two versions of the Behavioral Perspective Model (BPM), an extensional portrayal of socioeconomic behavior and a corresponding intentional approach, address the task of identifying where intentional explanation becomes necessary and the form it should take. This study explores a third version, based on neurophysiological substrates of consumer choice as a contributor to this task.

The nature of “value” is closely related to the rationale for a neurophysiological model of consumer choice. Value is the assignment of worth from the most positive to the most negative along a continuum that proceeds monotonically (Kahnt and Tobler, 2017). One dimension of valuation is represented by patterns of behavior. Animal activity, including that of humans, is marked by an approach to stimuli that increase the rate of behaving (reinforcers) and by avoidance of or escape from stimuli that reduce the behavioral frequency (punishers). Reinforcers are adaptive and positively valued while punishers are maladaptive and disvalued. A reinforcer is, therefore, something that an organism will work to obtain, a punisher something it will work to avoid (Rolls, 2019). This is the basis of what we may understand as behavioral valuation.

Hence, the extensional portrayal (BPM-E) treats consumer *behavior* as a response to the reinforcing and punishing consequences that similar behavior has previously produced. Reinforcing outcomes of purchase and consumption are assumed to increase the likelihood of the behavior in question being repeated, while aversive consequences are assumed to decrease that probability. The variables involved are operationally specified and measured with high intersubjective agreement. The intentional model (BPM-I), depicting consumer *action* in terms of mental processes such as perception, deliberation, and choice, extends the purview of the BPM to new situations and areas of explanation. BPM-E presents value as an intersubjective agreement reached by the parties to a transaction in the marketplace. BPM-I is concerned with the subjective valuation of what is to be given up in a transaction (usually money in the case of the consumer) and what is likely to be obtained (a product or service). Neuroeconomics proposes a third conception of value as the level of neuronal activity excited by the presentation of anticipated reward (Glimcher, 2009). As Padoa-Schioppa (2009) notes, the conceptualization of value in economics is behavioral and logical rather than psychological: since values are inferred from behavior, they cannot be measured independently of choice. It is, therefore, circular to assert that choice maximizes value. Such circularity is avoided if values are neurophysiologically computed: if a neural event correlates with a behavioral index of value, the former provides an independent measure of value, and the statement that consumers' choices maximize value becomes falsifiable. “For this reason, I view the discovery that values are indeed encoded at the neural level as a major conceptual advance and perhaps the most important result of neuroeconomics to date” (Padoa-Schioppa, 2007, 2009, p. 335; Glimcher, 2010; Serra, 2021). The capacity of this third conception of value to evaluate the extensional and intentional models motivates this study.

These models are employed within the intentional behaviorist research strategy, a three-stage methodology, comprising theoretical minimalism, intentional interpretation, and evaluation (Foxall, 2020). The first, extensional, stage ascertains whether it is feasible to portray the consumer as an idealized utility-maximizing system and how the utility involved is to be construed. Only when consumers have been shown empirically to maximize utility and the nature of what it is that they maximize has been identified, again empirically, *and* extensional explanation has been exhausted, is intentional interpretation justified. Failure of extensional explanation indicates the necessity of intentional interpretation and the functions it must fulfill. The intentional interpretation builds on empirical knowledge accrued during the theoretically minimalist stage, providing an intentional explanation based as closely as possible on an empirical basis and subject to a stringent evaluation. The third stage evaluates the intentional interpretation by its consonance with cognitive theory and its capacity to generate hypotheses that are testable by means of such extensional sciences as behavioral economics and neuroeconomics.^1^

## Consumer choice

“Consumer behavior” and “consumer choice” are often used interchangeably to denote the whole gamut of socio-economic behaviors involved in acquiring, displaying, and using products and services. Intentional behaviorism understands consumer choice in a more restricted fashion to denote situations where a consumer selects one of two or more options that have distinct implications in the short term and the long term.

Consumer *choices* are made when one of the options promises more immediate benefits than another, though these may be of smaller long-term value. Indeed, the selection of such a smaller-but-instantly-available item precludes the receipt of a greater good in the future. Speaking of consumer *choice* highlights customers valuing the easier-to-acquire but sooner-appearing item far more highly than what they know to be a superior product or service for which they must wait. It is possible that when they thought about these choices earlier in the day, they were fully resolved to hold out for the better option even though it required patience. However, the very appearance of the immediately available option, despite the knowledge that it is in some way or other the poorer alternative, raises its value dramatically (Ainslie, 1992). While many consumers yield to temptation under these circumstances, others exercise self-control, differing in the extent to which they “discount the future”, i.e., downplay the value of the delayed item simply because it is postponed. The impatient individual discounts “steeply”; the patient consumer, “shallowly”. Either way, conflict—between greater and less and between later and now—defines *choice*.

Accounting for choice solely in terms of the payoffs received (reinforcers and punishers) is a behavioristic interpretation, in which choice is determined by the outcomes it has previously produced and those currently on offer. These variables make the current choice predictable. Some outcomes strengthen responses that generated them and are known as reinforcers. Others, which weaken the responses, leading to a reduction in the rate at which they are performed, are punishers. An alternative mode of explanation is cognitivism which proffers desires, beliefs, emotions, and perceptions to account for actions. The emotional reactions of consumers to reinforcements and punishments may act as affective rewards and sanctions, respectively, and become the ultimate consequences of their consumer choices that influence the probability of their repetition. While reinforcers and punishers are empirically available events that alter the rate at which behavior is performed, the resulting affective states, subjective and private, are “rewards” or “sanctions”. This permits a final definitional clarification between behavior and action. “Behavior” is understood as the activity of an organism that is occasioned by events external to it, something that happens to the organism, which is passively involved in the process. Behavior is explicable, in terms of an extensional framework of conceptualization and analysis, as operant activity. “Action”, by contrast, is something that the organism does, originating within it, and with which it is actively engaged.

Our understanding of consumer choice, closely allied with temporal discounting, is concerned with the *current subjective value* of a future benefit, i.e., the value of that prospective reward rated by the individual consumer in the present moment. An individual's discounting behavior reflects their willingness to forgo a more immediate benefit in favor of a greater future benefit. Choosing the latter, larger reward (LLR) is said to embody self-control, while choosing the sooner, smaller reward (SSR) embodies impulsivity, and the balance between the two may indicate proneness to weakness of will, which at its most extreme may manifest as addiction. These are all loaded terms, of course, but attempting to understand them in light of valuing the future brings a modicum of rigor to the way in which consumption and addiction are understood.

**Figure 1:**
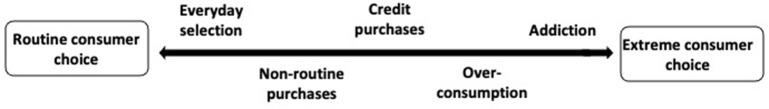
The continuum of consumer choice.

Consumers' choices range from the most routine and commonplace to the most extreme and compulsive. The choice of an everyday brand, a well-known and trusted item, exemplifies the first: there is a minimum of uncertainty and negligible risk. The opposite pole of the Continuum (Figure 1) locates more extreme consumption.

In between lie such activities as credit purchasing which expedites consumption at the cost of a higher final payment; despoiling the environment, e.g., unauthorized disposal of waste or the profligate use of finite resources, which may enhance the benefits of short-range consumption but subsequently impose larger outlays; and compulsive shopping which confers immediacy of ownership at a high price in the longer term. Except for some aspects of routine consumption, these activities require that the consumer pay more for temporal convenience: surrendering the future to immediate preferences. The idea of *consumer choice* encapsulates this tension between consumption at different times, each with its own outcomes, be they reinforcing or punishing, rewarding or aversive. What distinguishes them is the rate at which consumers discount the consequences of their activities.

## The intentional behaviorist research strategy

### Theoretical minimalism: BPM-E

The origin of the extensional model is the “three-term contingency” (Skinner, 1953), the essential explanatory device of radical behaviorism which defines the “contingencies of reinforcement and punishment” from which behavioral responses are predictable:


(1) $$\begin{array}{l} {\text{S}}^{\text{D}:}R\to S \end{array}$$

in which behavior is a function of antecedent discriminative stimuli (S^D^) which set the occasion for reinforcement and punishment (S^r/p^) contingent on the performance of a response (R). An additional source of antecedent stimulation takes the form of motivating operations (MO) which enhance the relationship between the R and the S^r/p^ (Michael, 1993; Laraway et al., 2003; Catania, 2013; for consideration of motivating operations in the context of consumer behavior analysis, see Fagerstrøm et al., 2010). A basic motivating operation might be, for instance, an enhanced impulse to consume food (R) if the consumer is in a state of deprivation. Taking MO into consideration, the “four-term contingency” is


(2) $$\begin{array}{l} \text{MO}:{\text{S}}^{\text{D}:}R\to S^{\text{r}/\text{p}}. \end{array}$$

**Figure 2:**
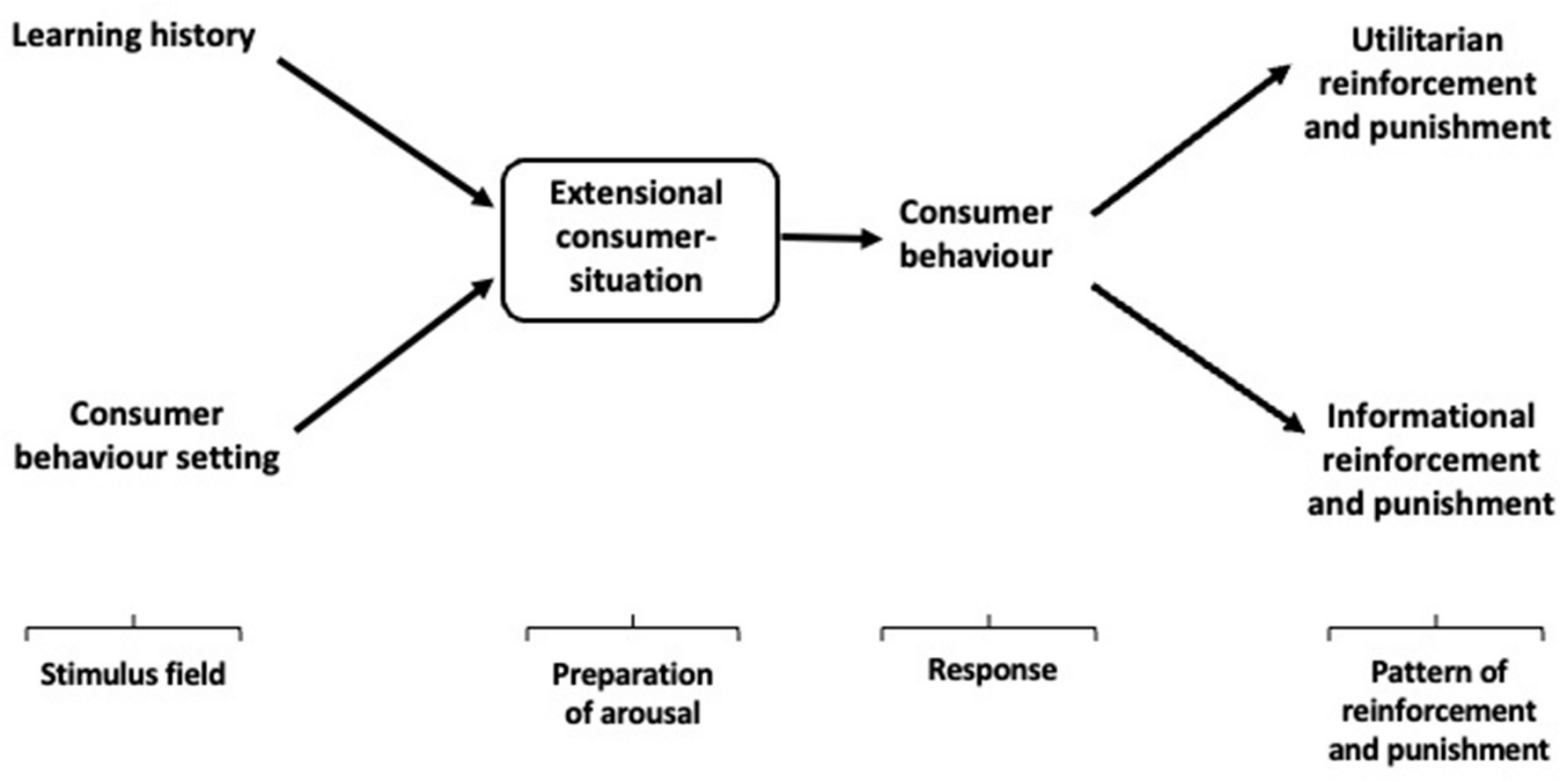
Extensional Behavioral Perspective Model (BPM-E).

S^D^s and MOs comprise the consumer behavior setting which, primed by previous similar behavior and its consequences (learning history), forms the consumer-situation (Figure 2). The consumer-situation is the confluence of temporal and spatial influences on consumer choice, the immediate precursor of consumer activity. In BPM-E, the consumer-situation is simply the interaction of the consumer's learning history and their current behavior setting. The synergistic effect of these influences, in which the learning history primes the stimulus field that composes the behavior setting so that its components become discriminative stimuli and motivating operations rather than neutral stimuli, is the immediate precursor and progenitor of consumer behavior. The primed stimulus field sets the occasion for the reinforcement of particular behaviors through the provision of utilitarian (functional) and informational (social) benefits. The consumer-situation, in this case, is conceived entirely as an extensional entity the elements of which can be objectively identified and measured; similarly, the pattern of reinforcement and punishment presented to the consumer as contingent upon their behavior is extensionally conceived and objectively specifiable.^2^

The extent to which a consumer behavior setting shapes consumer behavior is its *scope*. A relatively open setting contains many opportunities for a variety of behaviors, while a relatively closed setting restricts behavioral options to one or two. A party offers numerous ways of behaving and is relatively open in scope. “Open” does not mean *any* behavior will be tolerated but there is a gamut of options available to the consumer. Being in a formal church service, however, restricts behavioral possibilities and is relatively “closed”, and deviation from prescribed behavior may be punished. Between these extremes are many consumer settings that range in scope.

The outcome of the consumer's acting is the formulation of an inter-subjective exchange value, *V*_1_. Both buyer and seller concur in this evaluation, at least to the extent to which it reveals the price at which they were willing to exchange the good in question in an open competitive marketplace. *V*_1_ is a conceptualization of value as an intersubjective agreement based on the price at which an exchange has taken place. More formally, *V*_1_ is intersubjective value as established in the marketplace (Foxall, 2021). This is a socially constructed index based on collective intentionality: the understanding that the market is an institution that delineates the agreed worth of an item, that at which it is reasonable for it to be exchanged for a given sum of money or another commodity. This valuation can be established intersubjectively and is the socially agreed worth of the item based on exchange value. Insofar as obtaining this level of value in exchange, a relationship acts as an incentive to participate in a market exchange, and goods or money of this value constitute a reinforcer for this behavior. This concept of value is a mainstay of most neoclassical economic analyses including that provided by operant behavioral economics.

Some of the main antecedents of BPM-E are summarized in Box 1.

Consumer choice as considered in this model, as *behavior*, is determined by something that happens to the consumer, namely the influence of the consumer behavior setting in which they operate (or at least it can be described and explained by reference to a consumer-situation). Responsive behavior is not invariably impulsive, a single uncontrolled reaction to a set of circumstances. It may be considered not as a molecular response, therefore, but as a molar series of responses to similar stimulus fields, a sequence of behavior.^3^ And it is always responsive to a learning history, that is to the individual's previously enacted behavior of a similar kind and the reinforcing and punishing consequences it has engendered. It lies, nevertheless, at one end of a continuum of activities. In contrast to responsive behavior, considered action is the result of what the consumer does, their desires, beliefs, emotions, and perceptions (or which must at least be described and explained by means of this appeal to intentionality). Executive functioning, which leads to considered action, is relatively slow mental processing, with considerable working memory (WM) involvement, serial processing, decontextualized (abstract), requiring high cognitive level and processing ability, and reflective and learning capacity. Such action falls within the purview of the intentional model, BPM-I.

BPM-E has generated considerable empirical research relating consumer behavior to its pattern of contingent reinforcement and punishment in the contexts of product, brand, and store choice (Foxall, 2017a) which reveals what can only be known through the pursuit of an extensional approach. It also sets the limitations of such modeling, since an exclusively extensional perspective cannot account for aspects of the continuity and discontinuity of behavior,^4^ come to terms with the personal level of consumer experience, or delimit the behavioral interpretations of choice, necessitating thereby the intentional model, BPM-I (Foxall, 2020).

Box 1 **Antecedents of BPM-E.**

In tracing the foundation of radical behaviorism as a philosophy of behavioral science and a methodology for operant research, it is essential to consider the painstaking definition and delineation of the field by B. F. Skinner. Although not alone in this task (Skinner rather disarmingly pointed out on several occasions that he was not speaking as “*the* behaviorist”), his is a rigorous approach to an extensional science. Skinner (1938, 1945) sought to establish a *natural* science of behavior in which psychological terms refer to relations between the organism's behavior and the controlling environment in which it occurs (Morris, 2012). This science was founded upon the observable datum of a response occurring in an equally observable context composed of discriminative and reinforcing stimuli (Skinner, 1950). Radical behaviorism, as the philosophy of the behavioral science he developed, eschews reference to intra-personal psychic influences on behavior and seeks, not always successfully, to avoid intentional language. He was acutely aware of the implications of using intentional language for the adoption of intentional explanation. Even his last work was vitally concerned to delineate radical behaviorism from cognitivism based on linguistic usage (Skinner, 1989). His assiduity in defining verbal expressions to eliminate intentional explanation is exemplified by his allusion to the meaning of “in order to” as in the description of a fisherman spreading nets “in order to snare fish”. For Skinner, the “order” here denotes the temporal sequencing of spreading and ensnaring, rather than indicative of a purpose or plan for catching fish (Skinner, 1971).

### Intentional interpretation: BPM-I

**Figure 3:**
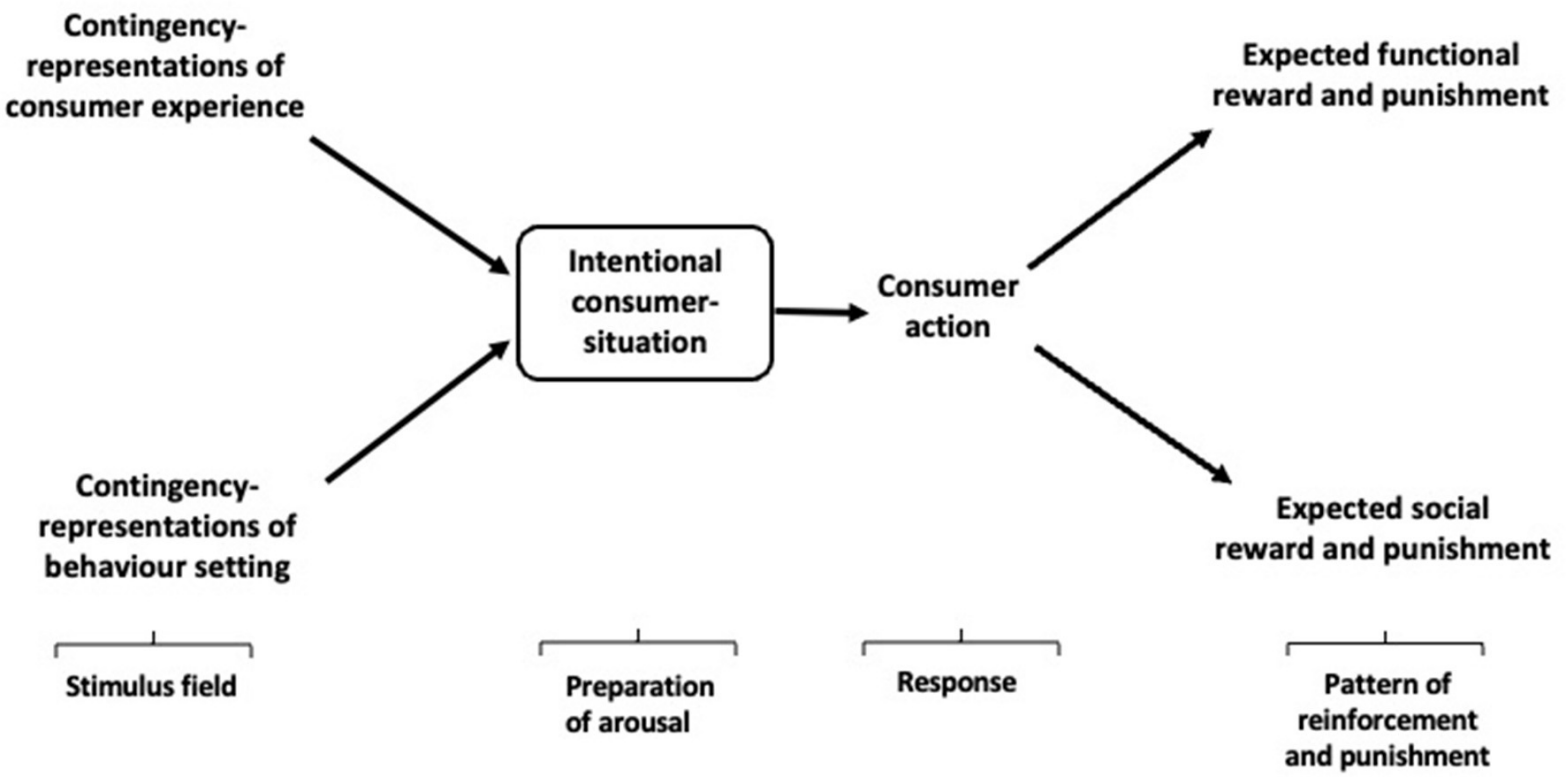
Intentional Behavioral Perspective Model (BPM-I).

In BPM-I, the intentional consumer-situation comprises the current consumer behavior setting as it is represented in the consumer's desires, beliefs, emotions, and perceptions (especially, as these refer to expected reinforcing and punishing outcomes of action), primed by their learning history as represented by their beliefs (Figure 3). The intentional consumer-situation therefore comprises (a) the contingency representations that inform and shape the consumer's action with respect to a set of contingencies of reinforcement that govern current and future choice, in light of (b) their perceptions of their consumption history, and (c) the beliefs which result from contemplation of that history and the probabilities of the reinforcing and punishing outcomes of further action.

Some of the key antecedents of BPM-I are shown in Box 2.

Box 2 **Antecedents of BPM-I.**

The foundations of BPM-I derive from two trends in psychological and philosophical work on the explanation of behavior. The first may be traced to behaviorists including Hull (1935), Tolman (1948), Deutsch (1960), Amsel and Rashotte (1984), Staats (1996), and Bandura (2022) employ intentional language and, thereby, intentional explanation. Despite what has been said about Skinner's care in linguistic usage, he came to freely admit that he used cognitive language to make clear his expositions of behaviorist explanation (e.g., Skinner, 1971, 1974). Other staunch radical behaviorists have found it equally necessary to employ intentional language to express themselves (Baum, 2017; for discussion of the implications of this, see Foxall, 2009). The problem with this is borne out by the second trend, an insistence by philosophers of the inevitability of intentional explanation when this apparently innocent attempt to communicate is employed. Among others, Chisholm (1957), Dennett (1969), and Taylor (1964/2021) have argued persuasively that such use of intentional language (that expressed in propositional attitudes containing “attitudes” such as *believes* and *desires* and content or proposition like “that it is raining”) necessarily entails intentional explanation and so departs from the mode of explanation on which radical behaviorism is based. Bridging these sources of influence, philosophically inclined psychologists such as Kimble (1996) elaborated stimulus–response behaviorism to embrace cognitive and other explanations.

The outcome of this consumer-situation, before the consummation of a purchase, is a subjective value, *V*_2_, which is the consumer's personal estimation of the worth of the item to be purchased, having both utilitarian and informational components. It exists only as an intentional object, accessible only to their, but nevertheless embodying the value they is willing to surrender to obtain the commodity. Whether it becomes objectified in the marketplace, revealing the commodity's *V*_1_ depends on the coincidence of the *V*_2_ values of the consumer and producer. “Coincidence” does not mean that the *V*_2_ values are identical: (a) there is no way of knowing this anyway and (b) it implies that the consumer's *V*_2_ is in some sense at least as great as the *V*_1_ that is the agreed exchange value, and that the producer's *V*_2_ is no lower than this objectified valuation.

*V*_2_ is subjective value existing only in the mind of the consumer, i.e., how the individual personally rates the commodity.^5^ It may be expressed in terms of a notional amount of money or goods—that quantity for which they would be willing to exchange it. It may be more ineffable than this: the personal worth it has even though the individual would never wish to exchange it.

### Evaluation: BPM-N

**Figure 4:**
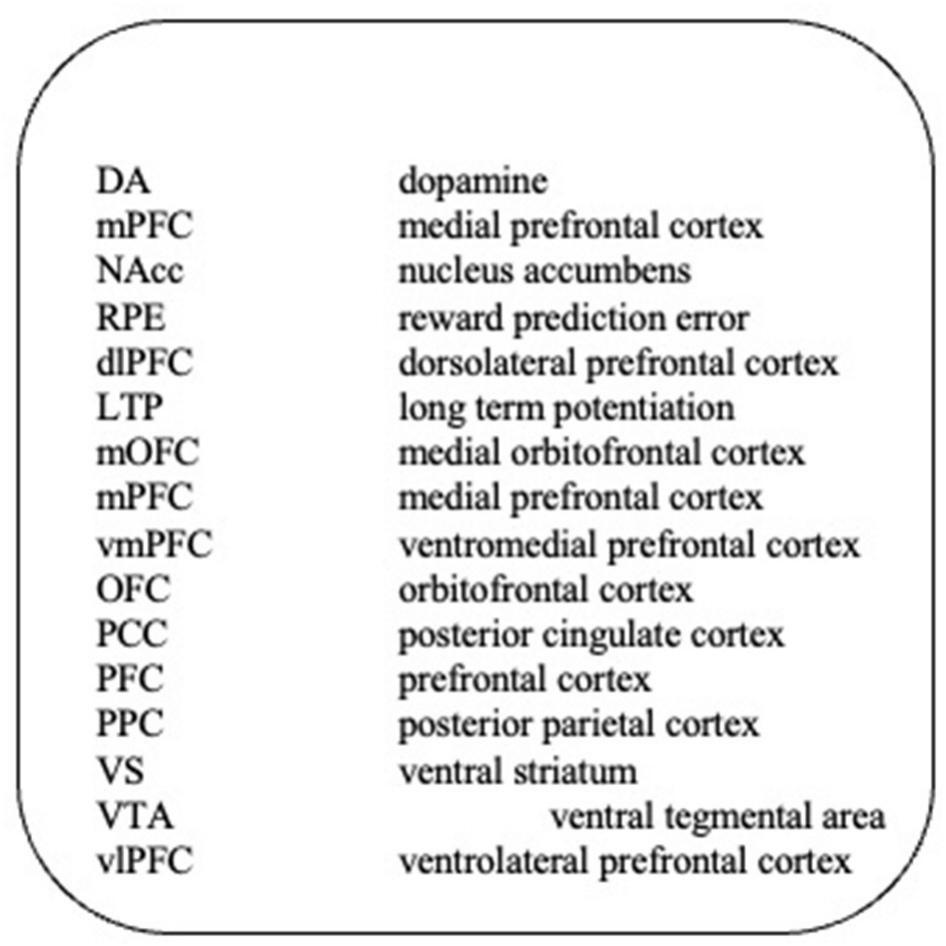
Abbreviations.

The third stage of intentional behaviorism is the evaluation of the intentional interpretation in the light of (i) its consistency with the empirical findings derived from the testing of the extensional model, (ii) its predictive capacity, (iii) rendering intelligible phenomena that are beyond the scope of the extensional analysis, (iv) its consistency with cognitive theory, (v) its capacity to engender hypotheses based on economic analysis such as those provided by operant behavioral economics, neuroeconomics and picoeconomics, and (vi) its consistency with neurophysiological evidence (Common abbreviations are listed in Figure 4). All but the last of these have been the subject of earlier work (Foxall, 2017a,b, 2020). The final criterion necessitates a neurophysiological model.

**Figure 5:**
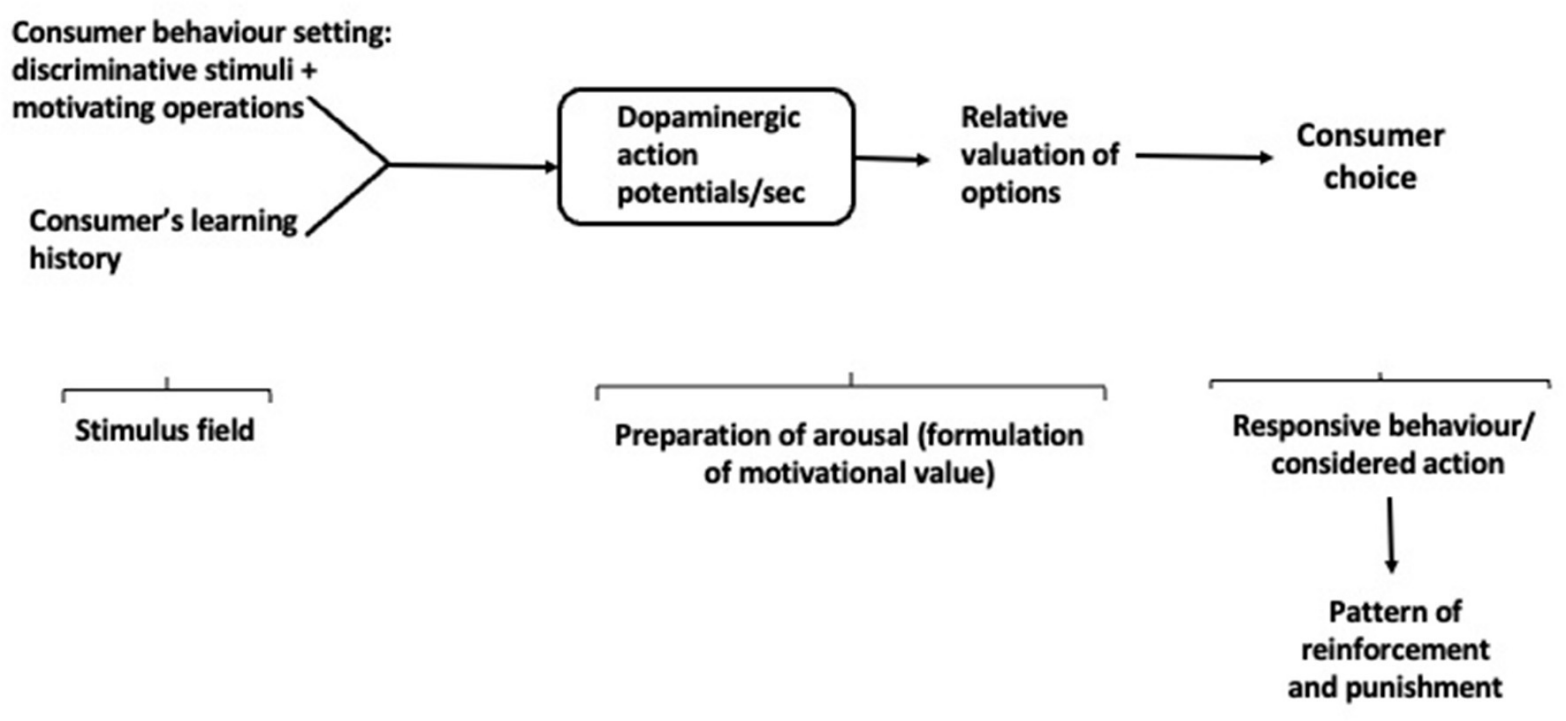
Neuropsychological Behavioral Perspective Model (BPM-N).

The extensional model provides a mechanism for establishing the values of commodities as they are intersubjectively determined in the marketplace (*V*_1_) while the intentional model treats the subjective values of consumers and marketers (*V*_2_) that guide market transactions. But BPM-E and BPM-I do not tell the whole story. There are also “objective” valuations, achieved in the course of the neurophysiological events that precede and accompany the creation of *V*_1_ and *V*_2_. This “neuro-valuation” composes *V*_3_, and the model which encapsulates its formation and operation is the neurophysiological model of consumer choice, BPM-N. It treats the role of DA in the reinforcement of routine and extreme consumer choice and interprets the rate of firing of dopaminergic neurons as a measure of valuation of the rewards which occasion them (Figure 5). Dopaminergic action potentials, the basis of *V*_3_, indicate how the stimulus field comprising the consumer behavior setting “sets the occasion for reinforcement”, in concert with the consumer's learning history.

#### Incentive salience

The consumer behavior setting offers a means of establishing relative values of reinforcers competing for the consumer's attention. The presentation of a reinforcer both physically and in thought, as well as the stimuli that predict its occurrence, is associated with the release of DA, which prepares the consumer for an appetitive response. Not only are S^D^s implicated in this increase in the rate of action potentials observed in dopaminergic neurons but DA is also implicated in raising the salience of situational stimuli previously associated with consumption and this further expedites excessive behaviors (Berridge and Robinson, 2011). Salience refers to whatever brings an item to the fore so that it stands out from others and its significance is that the brain “prefers” to process items highly salient in terms of color, orientation, size, pitch, and velocity (Horstmann, 2016; Foxall et al., 2024).

Reinforcers and rewards that regulate the frequency of behavioral responses are goals whose attainment increases biological fitness. Midbrain DA neurons innervate neurons in the VS and PFC in response to reinforcers and stimuli that predict them. Their phasic firing rates encode reinforcement predictions and establish reward prediction errors that relate not only to the acquisition of reinforcers and rewards locally but, thereby, to the enhancement of fitness. Reinforcers are processed in the VS, mPFC, and OFC. OFC is also involved in the desirability of a reinforcer compared with its alternatives. Reinforcers and punishers, which represent the individual consumer's comparative values, are indexed by the rate of dopaminergic action potentials and the probability and magnitude of the predicted reinforcers/rewards. OFC neurons relate reinforcers and punishers to prevailing intra-personal and extra-personal contexts determining the valuation on which decision-making rests are accomplished (Platt et al., 2017). DA-related activity in VS, amygdala, and OFC, areas known to intervene between sensation and action, contributes to the network through which valuation takes place. OFC, in particular, transforms reinforcement/punishment into a common currency of valuation.

#### Reward prediction error

The relationship between reward valuation and learning history is captured by “reward prediction error” (RPE), the difference between a reinforcer actually obtained and that predicted or expected. RPE may be negative (i.e., reward is predicted but not obtained) or positive (i.e., reward is not expected but is nevertheless obtained) (Schultz, 2010). The assumption that RPEs are reflected in dopaminergic neurons” firing rates is fundamental to neuroeconomics, as is the finding that whether an environmental stimulus engenders learning is not simply a matter of its presentation but is being unpredicted, novel, or surprising. The unpredictability of a reward is described by the *prediction error* term, λ – ΣV, where λ is the strength of association between a stimulus that fully predicts a reinforcer and ΣV is the joint associative strength of all signals present in a learning episode. Therefore, the prediction error, λ – ΣV, indicates the degree to which the reinforcer's occurrence is novel, surprising, unpredicted, or unexpected (Schultz and Dickinson, 2000). Learning depends on the acquisition of outcome predictions that are environmentally based (reinforcers or punishers) and/or internal states (emotional rewards or sanctions) (Schultz and Dickinson, 2000, p. 476; Rolls, 2019). Schultz and Dickinson (2000) propose a homeostatic principle by which behavioral outcomes that produce a mismatch (prediction error) between expected and actual reinforcers alter subsequent behavior, reducing the gap between outcome and prediction. Behavior modification proceeds until the prediction error reaches zero, i.e., the discrepancy between expected and actual reinforcement has been removed.^6^

#### Clarifying V_3_

Glimcher (2009) defines “subjective value” which approximates economic “utility”. It is operationalized as the number of action potentials per second, measured as mean firing rates of designated neuronal populations. To avoid confusion with *V*_2_, this is referred to here as neuronal/neural *value* and designated *V*_3_. Both Glimcher's idea and that of *V*_3_ are neuroeconomic conceptions of value. Empirical evidence, including fMRI results, suggests that neuronal value is encoded by the mean activity in the mPFC in the case of an action and the VS in the case of a good (Glimcher, 2010). Hence, *V*_3_ constitutes a metric of a physical event. The comparative valuation of alternative incentives rests on the degree of preparedness or arousal the dopaminergic reward system achieves in the form of action potentials. The incentive that occasions the greater rate of neuronal firing is thus valued higher. This is the reinforcer that will engender the greatest utility for the organism and optimally increase its biological fitness.

#### Evaluative role of BPM-N

How far does BPM-N elucidate the operation of the extensional and intentional models? This can be addressed by, first, considering BPM-E and BPM-I as components of a dual process theory (DPT) of consumer choice and, second, examining the implications of the neurophysiological model for this development. BPM-E and BPM-I form conceptual bases of the influences on consumer choice that dual process theories assume. The next section, first, appraises BPM-E and BPM-I as explanations of competing behavioral/action tendencies and, second, examines the continua established in the light of key dimensions of neural valuation.

### BPM-based dual processes theory

#### Working hypothesis

The working hypothesis is

that BPM-E constitutes a model of responsive–impulsive behavior, BPM-I one of reflective-executive action, a conjecture that may be examined via the contribution of BPM-N to the resulting DPT.

Its rationale is as follows.

While BPM-E is concerned with *behavior* responsive to a stimulus field and explicable without resorting to cognitive conceptions, BPM-I deals with *action* that arises from consideration of its probable long- as well as short-term consequences. This resonates with the numerous DPTs that capture the two broadly defined styles of reaction to the threats and opportunities presented by an organism's environment. These are (a) a rapid response that meets the immediate demands of the situation, a response attuned to the maximization of the organism's welfare fairly instantly and in line with its proximate circumstances, and (b) a measured reaction that takes more distal outcomes into account. The possibility arises, therefore, of treating these two models as depicting the competing tendencies of a DPT of consumer choice.

As far as consumer activity is concerned, the extensional and intentional models thus approximate the polar opposites assumed by dual process modeling. Responsive behavior, the domain of BPM-E, is the result of operant conditioning or innate action tendencies and is under the control of environmental stimuli operating in the context of learning history. Learning history is the sum of the consumer's past behavior and its reinforcing and punishing consequences. The environmental stimuli are of two kinds, S^D^s and MOs: together they form the stimulus field which, as it is primed by the consumer's learning history, forms the consumer-situation, the immediate precursor of consumer behavior. Responsive behavior is marked by its speed of operation, being the result of automatic mental processing, minimal use of working memory (WM), consisting of operations that occur in parallel, require little by way of cognitive level, and operating in specific (concrete) contexts (Stanovich, 2009). We can see this behavior as the immediate response to a situation of passing opportunity or inevitable threat. The system that produces responsive behavior is sometimes called the *impulsive system* and is responsible for sensation seeking, reward sensitivity, behavioral disinhibition, attention deficit, reflection deficit, and impulsive choice. In this case, the stimulus field, primed and activated by the learning history and emotional reactions, is responsible for the release of DA, arousal, and the preparation of the response. There being little or no reflection or inhibiting thought, the result is rapid response. The underlying behavioral mechanism can also be depicted in terms of operant conditioning. These mark out the activity that results as *behavior* in the sense of it being the result of what is happening and has happened previously to the consumer, i.e., *responding* to stimuli that occur in the current environment and the consumer's learning history. If there can be said to be an agential mainspring of this activity, it is apparently located in the environment.

*Considered action*, by contrast, is what the consumer does, outcomes of their desires, beliefs, emotions, and perceptions. The system responsible for considered action, the reflective-*executive system*, is based on attention, behavioral flexibility, behavioral inhibition, planning, and the evaluation of future events, and draws on WM. Emotions prompted by the consumer behavior setting are subject to self-regulation by the consumer and thereby tempered by reflection and experience. Metacognition, leading to further consideration of past behavior and its outcomes, enables consideration of probable consequences of current responses. These mark the activity of the consumer as *action* in that it derives from their desires, beliefs, emotions, and perceptions, reflecting active consideration of past behavior and its consequences, and imaginative projection of the outcomes of the current action. Guided by these executive functions, the consumer herself is the agential source of activity.

An extreme behavioral response may result from steep temporal discounting of rewards and thereby be impulsive. A relatively hyperactive impulsive system and relatively hypoactive executive system occasion a pathologically impulsive response in which an inferior reward that becomes available earlier is preferred to a superior reward that is delayed. Considered action, the result of relatively shallow temporal discounting, reflects—again at the extreme—a hyperactive executive system and a hypoactive impulsive system resulting in pathological response inhibition. In most instances, the interaction of individual differences in psychological and behavioral traits which are approximately normally distributed bring about the array of consumer choices, ranging from everyday consumption to addictive compulsion.

**Figure 6:**
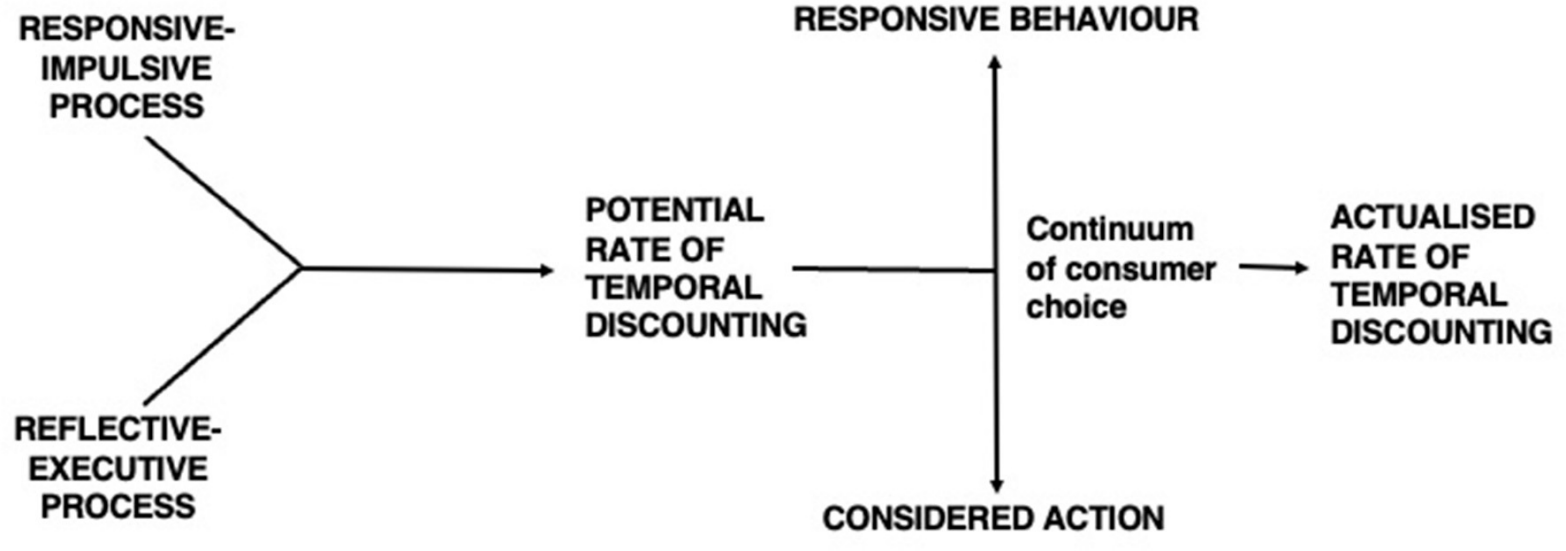
Determination of temporal discounting rate.

This is consonant with the competing neuro-behavioral systems model (Bickel and Yi, 2008; Bickel et al., 2020) which envisages the individual's temporal discounting rate as the balance among their cognitive, neurophysiological, and behavioral tendencies (Figure 6). The components of the BPM DPT as a responsive–impulsive process are strongly influenced by the limbic and paralimbic systems and a reflective-executive process is strongly influenced by PFC. The responsive–impulsive process stimulates immediate gratification and emphasizes behavioral outcomes that are reward-sensitive. The ensuing behavior is relatively uninhibited by longer-term consequences, utilitarian or informational. Behavioral consequences that fail to reflect non-immediate concerns are likely ignored. The impulsive choice is, therefore, emphasized and the future is steeply discounted. The reflective–executive process, however, attends to longer-term implications of action, relying, first, on cognitive and metacognitive operations which promote flexible behaviors, inhibiting those likely to produce deleterious consequences, and second, on WM and cognitive rehearsal to examine the consequences of the action that necessitate forward planning and responsibility for behavioral outcomes. It thus constrains the impulsivity of immediate and unconsidered responses.

The interaction of these processes determines the consumer's potential temporal discounting rate. Routine and extreme patterns of consumer choice result from peculiar concatenations of their consumption history and cognitive and affective appraisals of prior activity and that prefigured by the current consumer behavior setting. Routine consumer choice displays a rationality (cognitive or behavioral) marked by measured and predictable activity and expected results. Suboptimal (extreme) consumer activity reflects subjective overstatement of the rewards of consummatory behavior and preference for immediate satisfaction, the basis of reinforcer pathology theory (Bickel et al., 2020). The emergent pattern of activity, encompassed by the Continuum, reveals the actualised rate of temporal discounting achieved by the consumer.

### Neural valuation

**Figure 7:**
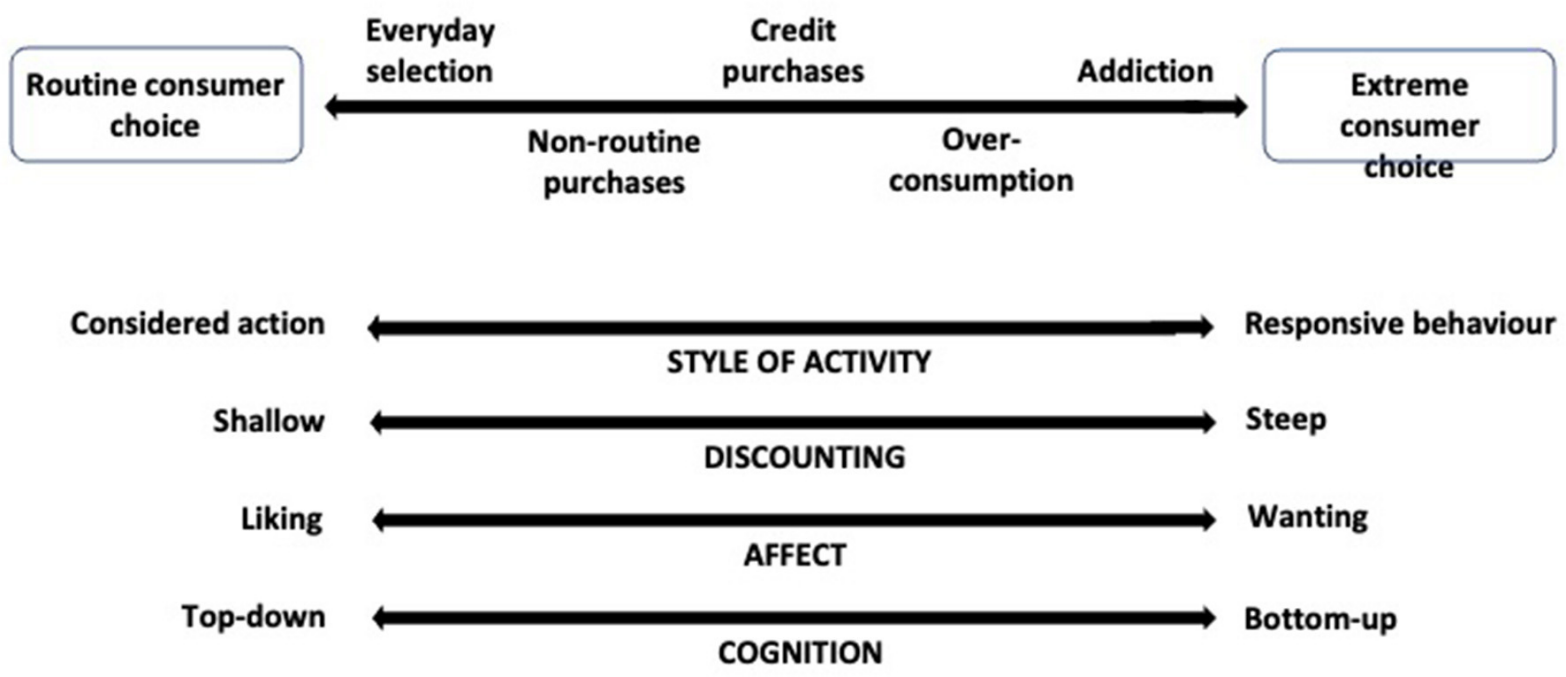
Dimensions of responsive behavior and considered action.

There emerge three key dimensions of the interaction of the responsive–impulsive and the reflective–executive systems: degree of discounting, affective tone, and cognitive procedures (Figure 7). A DPT ought to reflect differences in all of these, and we concentrate here on their neurophysiological bases.

#### Discounting

If patterns of temporal discounting depend on neurophysiological events, then brain regions differentially associated with decisions distinguished by their time horizons should be apparent. McClure et al. (2004) identified a brain region based on the visual cortex, premotor area, supplementary motor area, intraparietal cortex, right dlPFC, right vlPFC, and right lateral OFC, that is activated for decisions concerned with either immediate or delayed rewards. However, a second region based on VS, medial OFC, and medial PFC exhibited enhanced activity in the case of delayed reward. McClure et al. (2007) reported that the VS, subgenual cingulate cortex, mOFC, PCC, and precuneus exhibited greater activity when immediate rewards were involved rather than delayed rewards alone. There emerge “two sets of brain regions, one associated with presence of immediate gains and the other associated with presence of gains even in the absence of immediate options. Both involve brain regions associated with goal pursuit (prefrontal structures). However, the brain regions linked to presence of immediate gains also involve structures related to valuations (striatum) and cognitive control (cingulate and precuneus)” (Frost and McNaughton, 2017, p. 56).

The responsive–impulsive system incorporates the amygdala and VS, a midbrain region involved, through enhanced dispersal of DA during reinforcement learning, in the valuation of behavior. This is so for routine as well as extreme behavior: receipt of all kinds of positive reinforcers stimulates DA release in the NAcc. However, this midbrain region is inclined toward hyperactivity via “exaggerated processing of the incentive value of substance-related cues” (Bechara, 2005, p. 1459). In extreme consumption, drug-stimulated responses follow enhanced activity in the reward and valuation systems, the amygdala evincing especially amplified sensitivity to reward (Bickel and Yi, 2008). The process is engendered by either utilitarian reinforcers, in the form of drugs of abuse, food, and opportunities to gamble, or informational reinforcers such as social reward or self-esteem. Acquisition of money, which embodies both utilitarian and informational qualities, has the same effect. In drug abuse, such brain reward is acute: such substances occasion LTP at specific hippocampal synapses while the amygdala is concerned with the engraining of a learned (conditioned) response to the setting stimuli that accompany the use of the drug, notably social reinforcers and physical discriminative stimuli.

Bickel et al. (2012) note that both trait impulsivity and state impulsivity are found within the impulsive system. The former comprises behavioral regularities showing cross-situational resilience (DeYoung, 2013) and includes sensation-seeking (venturesomeness), related to optimum stimulation level and associated with sensitivity to reinforcement (Bickel et al., 2012). Substrates of trait impulsivity include mesolimbic OFC, mPFC, pregenual ACC, and vlPFC; sensation-seeking correlates with activity in right lateral OFC, subgenual anterior cingualate cortex, and left caudate nucleus activations. State impulsivities include behavioral disinhibition, attentional deficit impulsivity, reflection impulsivity, and impulsive choice, of which behavioral disinhibition is associated with deficiencies in ACC and PFC, attentional deficit impulsivity with impairments of caudate nuclei, ACC, and parietal cortical structures, as well as with strong activity in the insular cortex; reflection impulsivity follows impaired frontal lobe function; and impulsive choice accompanies increased activation in limbic and paralimbic regions during the selection of immediate over delayed reinforcers.

Processing relative reward values of alternative courses of action in the midbrain and prefrontal areas occurs predominantly through a feed-forward circuit that links the VTA via the VS to the OFC (Ross, 2011). However, changes in tonic DA concentration in the striatum affect general alertness of and receptiveness to chances of consuming rewards. However, phasic changes in the uptake of DA in the NAcc of the VS integrate components of reward functions including relative valuation, maintenance of attention, and preparation of motor response. DA relates the contingencies that predict reward with expected values thereof and this may present a severe problem: approximately half of 1% of populations who have been assessed possess dopaminergic reward systems that respond to frequent gambling by increasing tonic DA production on an ongoing basis (Ross et al., 2008; Ross, 2011, p. 57).

Such outcomes are not inevitable: they are mitigated by executive functions which promote anticipation of behavioral after-effects, reflecting pre-behavioral planning, foresight, and evaluation of reinforcing and punishing consequences of responding. The reflective–executive system, based in PFC, becomes hypoactive in extreme consumer choice. OFC may play a dominant role in temporal cognition by integrating reward magnitude and delay (Sosa et al., 2012). In the absence of such moderating effects, the impact of a hyperactive dopaminergic reward system is exacerbated, resulting in dysfunctional behavior. An impetus to such extreme behavior is provided by the augmented incentive value placed on such reinforcers as drugs, alcohol, food, and gambling, and the social and physical setting stimuli that predict them, as a consequence of amygdala-based processing of reinforcers, combined with impaired ability to inhibit behavior, the outcome of dysfunction of the frontal cortex (Bickel and Yi, 2008; Rolls, 2019; Bickel et al., 2020). Such imbalance marks extreme consumption.

Evidence implicates PFC in top-down self-regulation of brain regions such as the striatum which plays a role in reward processing and the amygdala which is similarly concerned with emotion (Kelley et al., 2019, p. 10). Interaction of PFC and the reward circuit determines the degree of self-regulation achieved (Heatherton and Wagner, 2011; Kelley et al., 2015). Self-awareness necessitates the operations of mPFC, damage to which presages impaired self-reflection and introspection. Prefrontal areas are essential for cognitively based executive functions that promote self-regulation, notably vmPFC, OFC, lateral PFC, and ACC (Heatherton, 2011, p. 368-−373). The inability of those suffering damage to vmPFC to control social and emotional activities (Damasio, 1994), whose insensitivity to social feedback and social norms leads to their continuing in asocial activity despite their awareness of this (Heatherton, 2011, p. 374), reduces their sensitivity to receiving and providing informational reinforcement. ACC is involved in the consciousness of the need to exhibit cognitive control necessary for self-regulation; damage to ACC is implicated in reduced awareness of conflicts and the need to exercise cognitive judgement to de-escalate them (Heatherton, 2011).

An important source of control over immediate responding in the face of environmental stimuli is cognitive processing to inhibit or alter emotional reactions. The affective reaction of the responsive–impulsive system, if unchecked by PFC-based cognitive regulation, influences rapid behavioral response. The inter-relationships of the amygdala-PFC circuit in the rapid generation of emotionally based impulses and the probability of their subsequent inhibition through cognitively founded self-regulation are, therefore, central to the conflict between the choice of SSR or LLR. The mesolimbic DA system, essential to the preparation of arousal, motivates the immediacy of response; the proficiency of cognitive processes in overcoming this determines whether the corresponding consummatory responses occur. There is no need for direct contact with reinforcers or stimuli predictive of them for this to happen: pictorial depiction of these stimuli or thinking about them suffices.

#### Affect

Despite the widespread assumption that “liking” and “wanting” are coterminous, they are dissociable both conceptually and in terms of the neurophysiological substrates that mediate them. Wanting, or incentive salience, results from “large and robust neural systems that include mesolimbic dopamine”, while liking, or hedonic impact, “is mediated by smaller and fragile neural systems, and is not dependent on dopamine” (Berridge and Robinson, 2016). *Contrary* to the notion that DA mediates pleasure, it is unnecessary for the hedonic experience. DA, rather, mediates desire or craving. Note that Berridge and Robinson employ wanting, without quotation marks, and “wanting” in distinct senses. The first refers to the ordinary usage refers to cognitive desire which has a declarative goal, i.e., an intentional state such as “I desire *that*…”. “Wanting” (in quotes) denotes desire or craving “mediated largely by brain mesocorticolimbic systems involving midbrain dopamine projections to forebrain targets, such as the nucleus accumbens and other parts of striatum”. “Wanting” is less connected with cognitively based objectives than cues for reward, which renders them especially conspicuous and appealing. The strength of the appeal depends on the individual's learning history and the structure of their neurophysiological response mechanism: “The intensity of the triggered urge depends both on the cue's reward association and on the current state of dopamine-related brain systems in an individual” (Berridge and Robinson, 2016, p. 671–2).^7^

Hedonic hotspots are responsible for “liking”^8^ contrast with the large, robust “wanting” system by being smaller and displaying a functional fragility. The interrelated pleasure hotspots that comprise the hedonic system mediate not only biogenic pleasures such as those obtained from normal eating but also the socio-genic pleasures of social and cultural intercourse. Both utilitarian and informational reinforcement, therefore, derive from this source. The hotspots are, moreover, located within much larger limbic structures—tiny, highly circumscribed in the neurochemistry they are responsible for, and easily disturbed—properties leading Berridge and Robinson (2016) to suggest that intense pleasures are rare in animals and humans compared with intense desires.

Principal locations of hedonic hotspots are limbic PFC, orbitofrontal, and insula regions, which in humans code sensory and higher pleasures, along with other deep subcortical brain areas. They function, stimulated by opioid or endocannabinoid neurotransmitters, to exacerbate “liking”, making sweetness, for instance, pleasanter. Yet, DA-based stimulation of hedonic hotspots fails to enhance “liking”, as its role is confined to “wanting” (Smith et al., 2011; Berridge and Kringelbach, 2015; Berridge and Robinson, 2016). Berridge and Robinson (2016) draw special attention to a hedonic hotspot found in ventral palladium (at the base of the subcortical forebrain), which intensifies “liking” for high levels of pleasure and also, when minimally lesioned, eradicates normal pleasure and overturns the hedonic experience of sweetness from “liking” to “disgusting” (Peciña et al., 2006; Robinson et al., 2013; Ho and Berridge, 2014; Berridge and Kringelbach, 2015).

“Wanting”, then, is mediated mostly by the mesocorticolimbic system in which DA produced in midbrain VTA is delivered to the forebrain, notably the striatum including the NAcc. Berridge and Robinson (2016) point out that the intensity of the stimulus to behavior that is thereby generated is a function of the cue's association with reward (the consumer's learning history as “recorded” in its neurophysiology) and the state of the individual's dopaminergic neurons. They further note that “This interaction allows ‘wanting' peaks to be amplified by brain states that heighten dopamine reactivity, such as stress, emotional excitement, relevant appetites or intoxication”. Because of this amplification, addicts struggle to limit consumption. Hence, addiction is less a matter of satisfaction, pleasure, need, or withdrawal than of “wanting”.

Amplification of incentive salience exacerbates addicts' continuing to consume: initial use of drugs/behavioral rewards inspires increased DA production which eventuates in craving. The consumer's learning history may, therefore, engender a propensity to addiction such that the cognitive desire to quit drugs cannot diminish their incentive salience. Progressing from routine to extreme consumption, liking diminishes while wanting increases. Routine consumption manifests cognitive and emotional control; and extreme consumer choice, immediate gratification, and automatic response to stimuli preclude cognitive intervention. Consumers, even when conscious of how their behavior is governed by environmental rewards, persist in it. The dual interactive systems are, then, responsive–impulsive behavior, with or without awareness, and executively controlled action. Through sensitization—a disproportionately strong response to a stimulus, in which the consequences of consumption result in an enhanced or exaggerated effect—the “wanting” system becomes hyper-reactive not only to stimuli such as drugs or other substances, like food, that have acquired intense incentive salience but to stimuli that predict them like cues and contexts; hence, contextual stimuli acquire incentive salience in themselves, making consummatory behavior more probable, more intensely rewarding, and more strongly reinforced. Moreover, while “wanting” is exacerbated by sensitization, “liking” is not, and may actually decline as “wanting” surges. Such wanting is, moreover, persistent (Berridge and Robinson, 2016).

Consumer behavior settings thereby become more desirable and more behaviorally determinative. The stimulus field becomes a source of reinforcement in its own right while the discriminative stimuli and motivating operations that compose it become surer precursors of consumption. To the behaviorist qua behaviorist, it is sufficient to point out that this is the case, since behaviorism is interested only in the behavior itself and its extra-personal context, the super-personal level of exposition. To the cognitivist qua cognitivist, however, personal-level desires, beliefs, emotions, and perceptions deserve consideration since even the private (mental) perusal of the contexts in which reinforcement has previously occurred stimulates further appetitive and consummatory behavior. Interpretation of these processes is the business of BPM-I, but full comprehension requires an understanding of the sub-personal level, neurophysiology of reinforcement, affect, and conation, i.e., BPM-N.

#### Cognition

Decision-making is the process in which opportunities for reinforcement and reward are evaluated according to goals, leading to the selection of apparently optimal action.

Distal goals of behavior are phylogenetically shaped: reinforcers and rewards that regulate the frequency of behavioral responses are goals whose attainment enhances biological fitness. Rolls (2019) links emotional rewards and reinforcing contingencies: emotions are generated by instrumental reinforcers. Broad primary (biological) goals of behavior are influenced by genes that “selfishly” regulate what will act as primary reinforcers and punishers to promote their survival through the biological fitness of organisms that are their vehicles (Dawkins, 1976). Although genes specify general adaptive goals of behaving by determining what can act as primary (utilitarian) rewards, they do not fix the specific behaviors or the secondary reinforcers that generate them (Rolls, 2019). For human consumers, secondary reinforcers emerge through experience, especially via social interaction that maintains informational reinforcement. These proximal goals of action reflect ontogenetic development. Analysis based on the BPM indicates close relationships between the contingencies of reinforcement and punishment and reported emotions (Foxall et al., 2012): utilitarian reinforcement is associated with pleasure, informational reinforcement with arousal, and the scope of the consumer behavior setting with dominance. Consumer activity is directed toward the maximization of contingent reinforcers and ensuing affective rewards, and minimization of punishers and affective sanctions. Consumers set strategic objectives, within the framework of evolutionarily, and operantly sourced objectives derived from their unique consumption histories: preferences for products and brands, retail outlets, magnitude and timing of purchases, and so on.

Corresponding to the styles of consumer activity outlined above, picoeconomics (Ainslie, 1992) describes and analyses the strategic interaction of competing interests within the individual. Picoeconomics is an economic psychology of the personal level, the domain of BPM-I. The first strategic tendency Ainslie identifies is “short-range interest” (SRI) that seeks rapid satisfaction, prompting behavior that exhibits steep temporal discounting, and akin to Freud (1900/1953) “primary mentation” (Brakel, 2009; Foxall, 2017b). The second is “long-range interest” (LRI), deferring gratification to enhance enduring wellbeing. The resulting action discounts shallowly and approximates Freud's “secondary mentation”.

Cognitive processing—maintaining attention, retaining information in WM, and hypothesis testing in disengaged imagination—entails mental labor which is minimized by attending to, memorizing, processing, and learning only what is most valuable. Encountering a new consumer behavior setting, the consumer attends differentially to salient features, scanning the environment for potential reinforcement and reward, and weighing the costs of securing them. Maintaining attention over the decision process is expensive. The responsive–impulsive system, pursuing SRI, provides the less exacting alternative since its bottom-up functioning makes it fast, stimulus-sensitive, and automatic, responsive to the proximal situation. Using the top-down LRI-sensitive executive system is, however, effortful, slow, and must be constantly cognizant of goals. Attention is allocated to the most salient stimuli and situations that have the greatest potential values (encouraging approach) as well as the least valuable (prompting avoidance). Top-down processing is the sphere of BPM-I; bottom-up, that of BPM-E; in the process of learning, the values of actions and states are ascertained via reward processing; and in the case of learning from prior transactions, consumers process *V*_1_ values which eventuate in the formation of *V*_2_ values.

Subjective valuations over a sequence of decisions are coded by the lateral intraparietal cortex, LIP (Hélie et al., 2017). In monkeys, LIP allies strongly with the identification of and attention toward valued locations and, conceivably, building “priority maps”—presumably a hierarchy of *V*_2_ values—which regulate top-down attention. The values employed in bottom-up processing derive predominantly from the species' phylogenetic history, focusing on places and stimuli (discriminative stimuli and motivating operations) that enhance survival and biological fitness. Insofar as they are hardwired, they remain relatively untouched by reward processing during ontogenetic development. Values impinging on bottom-up processing may be represented in the superior colliculus, important for eye movements and associated with LIP.

WM competence relies on attentive capacity. Neurophysiological correlates of WM are found in lateral PFC, thalamus, striatum, and ACC. Reinforcement learning based on dopaminergic neurons determines learning what is salient and consequently the contents of WM. Hence, the contribution of WM to valuation reflects reward maximization and reward processing. More particularly, our knowledge of RPs indicates, in the case of phasic DA, a reduced rate of action potential if an expected reward fails to occur and increases when an unexpected reward is received. Reward processing leads to the revaluation of the elements contained in WM through the alteration of neural plasticity. WM is *cognitive* and therefore intentional: its contents are intentional objects relating to events and behaviors, though they have neurophysiological correlates. WM is not a neural processor of information, however, it retains the most highly valued and salient items and eliminates the rest, but it is insufficient to cope with the cognitive demands of value-related reinforcer choice. This calls for LTM, an enduring store of retrievable information which is costly for cognitive effort and neurophysiological energy. Only if memory retrieval produces greater decision efficacy is it rewarded, and vice versa. The cost–benefit analysis involved must calculate the value of memory use, i.e., magnitude of potential gain from retrieved information and the likelihood of its effective deployment. LTM encoding of information involves the hippocampus, dlPFC, and PPC; retrieval, dlPFC, and OFC (Hélie et al., 2017). Valuation is key to what is encoded in and retrieved from LTM.

This valuation requires attachment of significance to competing activities and their outcomes given the consumer's goals. The presence of DA in the midbrain and initial prefrontal areas indicates “basic relative reward value computation” (Ross, 2011, p. 57). The principal circuit involved links VTA, VS, and OFC. Tonic changes in DA levels in the striatum promote alertness to any opportunity to consume a reinforcer presented by the external environment. The appearance of a stimulus, consumption of which is associated with reinforcement in the organism's learning history, engenders phasic reception of DA in the NAcc, a component of the VS responsible for integrating properties of reinforcers like their relative values, and for maintaining attention on the reinforcer and the opportunity to consume it, as well as initiation of appropriate motor responses. The dopaminergic reward system thus links environmental stimuli previously associated with reinforcement and expected values of the available rewards (Ross, 2011).

Decision-making is enhanced by a neurophysiological common currency enabling the values of commodities or courses of action to be compared. Valuation enables choice selection *and* post-consumption assessment of outcomes of consumer activity. Hélie et al. (2017) distinguish *valuation* (occurring before action) and *reward processing* (occurring afterwards), both of which are fundamental cognitive functions. The value of an action's consequences inheres in the reinforcement and reward they have led to, and such valuation is reflected in the immediacy and magnitude of these aftermaths of action: anything that reduces them is disvalued. Hence, central to valuation is temporal discounting, identified earlier as the hallmark of consumer choice. Valuations are subjective, indicating the consumer's perceptions of the current behavior setting in light of their learning history. Although the rate at which the consumer devalued the future is obvious *post-behaviorally*, the motivating factor is their pre-decisional subjective valuation (both cognitive and neurophysiological). Relevant common-currency valuations are probably located in vmPFC, VS, PCC, and, less so, amygdala, insula, and PPC (Hélie et al., 2017, p. 34).

Choice selection stems from information handling involved in the comparison of alternatives and picking out the one most likely to optimize. If the likelihood of each option's optimizing reward has been subjectively calculated (both neurophysiologically and cognitively), the consumer's utility function encapsulates the consumer-situation which is the immediate precursor of their activity. These valuation processes, the essence of decision-making, derive from neurophysiological valuation in mPFC, VS, PCC, amygdala, insula, and PPC. Prior learning contributes to pre-behavioral estimation of the values of states and actions which permits maximization of overall utility. Operant conditioning is central at the behavioral level, where the framework of conceptualization and analysis is BPM-E; if cognitive learning is emphasized, the conceptual frame is BPM-I; the establishment and implications of synaptic strength as a guide to learning at the neurophysiological level requires BPM-N.

Flexibility required during evaluation and choice inheres in the ability to switch from task to task, inhibit certain responses, and maintain information through WM. Also required is a cognitive arena in which mental rehearsal of future courses of action occurs without its speculative nature being mistaken for reality; the ability to perform such appraisals of multiple hypotheses “off-line” reflects the sagacity to distinguish different kinds of propositional attitude, notably the discrimination of reality-tested beliefs-proper from neurotic beliefs, suppositions, and fantasies (Stanovich, 2009; Foxall, 2017b). Such metacognitive rationality underlies executive consideration and action control. This intellectual activity—expensive cognitive procedures involving reasoned evaluation, planning, problem-solving, and decision-making—depends on anterior and lateral areas of the PFC. The principal areas are the anterior cortex and dlPFC, with lesser roles for ACC, PCC, temporo-parietal junction (TPJ), and PPC (Serra, 2021). Reward processing enhances the effectiveness of sequential decisions by enabling the subjective value of each option to be learned and its realized utility determined and lodged in LTM as a reference for future choice processing.

### Explanatory role of conceptual dual processes

#### Routine consumer choice

The most routinized example of consumer choice, everyday brand, product, and store selection is apparently accommodated fully by radical behaviorism: it surely depends only on the automatic response to the stimulus field presented in the form of a marketing mix. There is good evidence that this behavior is adequately modeled by BPM-E (Foxall, 2017a). The behaviors in question can certainly be understood and predicted as stimulus-bound responses, though they are hardly impulsive. We conclude that BPM-E is not confined to the responsive–impulsive system: it is clearly appropriate precisely where the reflective-executive system is expected to hold sway. This requires explanation. We might, for instance, see routine consumer choice as the outcome not simply of a stimulus–response sequence but as resulting from a deliberative procedure that brands have to go through before they find a place in the consumer's consideration set. Consumer choice has been portrayed as a three-stage progression comprising (i) Awareness, (ii) Trial, and (iii) Repeat-buying (Ehrenberg, 1988). The intensive advertising necessary to establish a new brand in an existing product category has the limited function of stimulating *trial* among a subset of purchasers of that class. An element of this can be seen solely in terms of response-to-stimulus given that the composition of the new brand may well be similar to that of existing brands. It is easy for the consumer simply to try the new brand which is already half-familiar. However, we may also see this as an instance of deliberation being also at work since the perception of the stimuli presented by the new marketing mix and the judgement that they represent an acceptable member of the product class is required. The radical behaviorist who is concerned only with observed patterns of behavior will not be impressed by this of course since they are not interested in explanation that goes beyond description; but cognitive psychologists, who understand behavior to be the outcome of mental processing of information, will be inclined to see this accommodation of a new commodity in terms of it being the outcome of a deliberative process.

This interpretation is supported by the trial and repeat stages. While only a relatively small sample of product-category users try a new brand—perhaps those who are heavy consumers—only a small subset of trialists become repeat-purchasers: those who deem the new item to be a satisfactory future member of their consideration set, a brand that can be relied upon to deliver the standard characteristics that must be evinced by a member of the product category. This is definitely a matter of deliberation and judgement which entails all of the cognitive procedures discussed above. The intentional explanation is also indicated insofar as the novelty-seeking that is entailed in the incorporation of a new brand into the consumer's consideration set and is explicable in terms of their personality traits—venturesomeness and sensation-seeking (Foxall, 1995).

We are fully justified, therefore, in viewing the routinized buyer behavior that we have labeled everyday brand choice as a proper focus of reflective–executive theorizing. This in no way removes BPM-E from its explanation—the patterns of choice observed in the research summarized in Foxall (2017a) are most appropriately designated operant and this layer of explanation adds much to an exclusively intentional approach. More importantly, it suggests a valuable symbiotic relationship between the extensional and intentional models which would be overlooked by a DPT that was insensitive to the empirical detail of consumer choice. Perhaps, a (sequential) combination of BPM-E and BPM-I is appropriate here: first, in dealing with the routine influence of a consideration set on patterns of choice BPM-E is invaluable but accounting for its composition entails cognitive considerations; second, while elements of both BPM-E and BPM-I are required to account for the initiation of the trial, the decision to repeat-buy, to incorporate the trialed brand into one's continuing consideration set, is something that depends on judgement and therefore BPM-I.

The more the new item deviates from the stimuli that define the prevailing produce category, the less able is the BPM-E to cope with the explanation of the discontinuity involved, and BPM-I becomes more relevant. Discontinuous innovations are by definition not routine as they are maximally disruptive of patterns of consumer behavior. Adopting them requires consideration, as does even trialing them in view of their expensiveness and embodiment of risk.

#### Extreme consumer choice

Extreme consumer choice, which entails steep temporal discounting, invites explanation in terms of BPM-E: it is stimulus-bound and predictable based on learning history and the current consumer behavior setting. But addiction is not necessarily the final resting place of the compulsive consumer, for whom recovery is a perfectly feasible option. Recovery, however, is likely to require cognitive intervention, requiring understanding in terms of BPM-I. All of the strategies for changing extreme consumer behavior proposed by Ainslie for forestalling the deleterious effects of addictive behavior require intentional explanation.

Ainslie (1992) speaks of conflicting *interests* which are concerned, respectively, with securing long-term benefits and short-term pleasures. The preference reversal which eventuates is characteristic not only of addiction but every day switches of preference that mark less extreme behavior. One of the strategies described by Ainslie, “bundling', requires a comparison of the cumulative benefits of a series of later-appearing rewards with the sum of immediate benefits of an immediate inferior choice, which allows the temptation to sub-optimize (through “willpower” or “self-control”) and to be overcome. Bundling, in common with other strategies Ainslie discusses, involves metacognition: not simply the cognitive effort involved in imagining future behavioral consequences, but the conjectured amalgamation, at a time when none of these consequences has been delivered or experienced, of the sum totalities of SSRs and LLRs, and their comparison. This necessitates comprehending how the first subsequent choice relates to the sequence of further choices, perceiving that the initial choice predicts later choices, and that the realization that making a choice either to take the SSR or defer gratification, entails pre-commitment to a future course of behavior.

BPM-E captures a portion of this behavior but does not tell the whole story of how the modification of choice comes about. The perceptual and cognitive processes implicated require an intentional account.

#### Intermediate consumer choice

The forms of consumer choice that lie between routine and extreme on the Continuum involve degrees of temporal discounting, albeit not of the steepest variety. *Non-routine* consumption such as the purchase of consumer durables has many characteristics of the discontinuous behavior entailed in the consideration and adoption of a new brand. Its description in operant terms is possible but it remains a discontinuity that may be fully comprehended only by reference to premeditation, the weighing of costs against benefits, and the comparison of alternatives. BPM-I is likely to predominate in rendering this behavior intelligible. Obtaining *credit* may also be understood to a degree in operant terms but, again, calls for considerations of cognitive calculation, the comparison of alternatives, and selection of a workable option based on its conjectured rather than experienced benefits. Cognitive decision-making is implicated, though the beginnings may lie in perception. *Environmental despoliation* often arises from the expediency of managing waste cheaply and rapidly, which gives rise to its consideration as a form of operant behavior. Increasing numbers of consumers are, however, concerned about the longer-term outcomes of such behavior and such environmental concern must be considered the result of cognitive consideration rather than the first-hand experience of the negative results of failure to protect natural resources. Environmental concern may, of course, be prompted by acquaintance with stimuli but it is largely a cognitive matter. This is a midway pattern of behavior that may be prompted by the apparently cost-free nature of acquiring some goods that require non-renewable resources to be consumed or of divesting oneself of goods no longer required. Therefore, BPM-E is highly relevant. However, the inauguration of an environmentally friendly style of consumption is likely to be preceded by prior cognitive deliberation which brings BPM-I to the fore. Finally, overconsumption, which may be connected closely with environmental despoliation, is a matter of behavior coming under stimulus control as the availability of reinforcers both utilitarian and informational expands and the means of satisfying cravings keep pace. This is clearly behavior under the control of fairly steep discounting and this tendency is more marked as we move from say over indulgence in foods and alcohol to compulsive purchasing. BPM-E is indicated as a means of explaining this behavior, though attempts to modify it may need to rely on perceptual and cognitive considerations and BPM-I.

#### Summing-up

Our working hypothesis has been that BPM-E is a model of the impulsive system displaying automaticity in the quest to satisfy short-range interests, while BPM-I deals with executive control and analytical deliberation pursued to attain a long-range interest. The analysis indicates evidence for this insofar as patterns of activity, discounting rates, affective reaction, and cognition portrays routine consumer choice as behavior that is impulsive, short-term, stimulus-bound, automatic, and thoughtless—traits well captured by radical behaviorism—and extreme consumer choice as an action that is considered, longer term, and intellectually based. These descriptions emerge from the contemplation of the kinds of pre-behavioral and behavioral spontaneity, even impetuousness, implied by a behavioristic model like BPM-E and the reflective and ruminative action, guided by its likely long-term consequences, implied by a model based on cognition and rumination like BPM-I. They are fully consistent with the theoretical principles devised by the authors of these and other DPTs, which rest in their turn on large volumes of empirical research in psychology, neurophysiology, and economics. This study, comprehended by the intentional behaviorist research programme (Foxall, 2016a,b,c, 2020), is underpinned by the empirical findings of neurophysiological research which are the province of BPM-N. This is generally consonant with the working hypothesis: the case for the DPTs of behavior/action made by a variety of authors seems justified. However, it is clear that the pattern these authors describe proceeds at a rather comprehensive level of interpretation. The next task is to ascertain whether this generalized picture might be fine-tuned in the specific context of the economic psychology of consumer choice. In particular, does this general conclusion oversimplify? Are there subtleties to the general pattern suggested by the working hypothesis? Is BPM-E *just* about responsiveness-impulsivity, and BPM-I *just* about reflection-executive control? Are the conceptual and explicatory properties of these models exhausted by their application to a single mechanism of behavior/action?

BPM-E does not inevitably imply automaticity: while not embracing cognitive contemplation, it comprehends verbal behavior insofar as language consists of discriminative stimuli and motivating operations. While it remains entirely an extensional, behaviorist explanation, its relevance may extend beyond the automaton-like and uncontextualised reaction of an organism to a set of passing circumstances. Operant behavior relies on the consumer's learning history and state variables, and it accommodates deleterious outcomes of unconsidered behavior. This argues for its responsiveness not only to the passing reward of instant gratification but also to the avoidance of outcomes that encourage irresponsible behavior. So, while BPM-E deals with stimulus-bound impulsive responding, it is not confined thereto, a response mechanism governed by automaticity, spontaneity, and unconstrained impulsivity reflecting the contingencies of the moment. Its explanations remain extensional, contextual, and circumstantial, but it is not exclusively relevant to activities that steeply discount the future. Radical behaviorism does not deny the existence of thinking and deliberation; without being specific about what they are, and without using them other than peripherally in its accounts of behavior, it acknowledges them as behaviors under operant control (Skinner, 1974).

BPM-I is similarly complex in the range of action to which it is relevant. To be sure, it provides an understanding of the structure and operation of deliberated action highly reliant on executive functions. Yet, while BPM-I is largely a source of explanation of the reflective–executive system, it is not confined thereto. The reflective–executive system is instrumental in shaping a form of behavior that is intermediate between responsive behavior and considered action, that which is essentially impulsive but which occurs with the individual's full awareness. While fully cognizant of the consequences of their actions, the compulsive consumer may engage in it regardless: we may think of this as “impulsivity with awareness” or “conscious impulsivity”. While the form of the behavior enacted falls within the realm of BPM-E, which considers it as a function of the stimulus field that comprises the consumer's behavior setting, its interpretation requires the understanding supplied by BPM-I.

These considerations identify why a conceptual DPT is required rather than one based naively on multi-componential units that seek to integrate behavioral, cognitive, and neurophysiological operations as though these did not rely on antithetical modes of explanation. They also indicate the necessity of any DPT to rely less on generalities about the spheres of activity to which they are applied. Rather, they should be tempered by and responsive to what is known about the particular domains of conduct to whose explication they are expected to contribute. In particular, what should be the “conceptual spread” of our efforts to explicate the range of consumer activities described by the continuum of routine and extreme consumer choice? That is, which models are actually relevant to the elucidation of routine and extreme consumption?

Familiarity with the observed patterns of consumer choice suggests that while the hypothesized allocation of explanatory resources proposed in the working hypothesis is generally accurate, important aspects of consumer activity over the Continuum require both BPM-E and BPM-I for their full comprehension.

## Conclusion

By introducing a neurophysiological model to the BPM suite, this study has sought to elucidate further the extensional and intentional explanations of consumer choice, especially in terms of the contrasting conceptions of value they present.^9^ The portrayals of consumer choice provided by the extensional and intentional models differ in their neurophysiological implications: the neural foundations of the discounting styles they suggest and the roles of affect and cognition in accounting for decision-making and activity. This in turn supports the possibility that BPM-E and BPM-I form the explanatory axes of a *conceptual* DPT of cognitive structure and function. Most DPTs are concerned with interpreting automatic and controlled mentation as distinct, albeit interrelated, kinds of cognition or metacognition. The proposed BPM-based DPT is, by contrast, concerned with the kinds of explanation appropriate to responsive behavior and considered action without portraying either of these styles of consumer activity uniquely in extensional or intentional terms. Rather, it points to the need to deploy both models to explain routine and extreme styles of consumer choice. Although the former is predominantly accounted for by BPM-I, the latter predominantly by BPM-E, there is scope for reversing this convention when the subject matter requires it.

The incorporation of a neurophysiological model, BPM-N, allows the differences in consumer activity assumed by the Continuum to be ascertained by reference to an independent measure of value, one which can be subjected to empirical appraisal (This conception of value is “objective”: in contradistinction to behaviorally derived super-personal intersubjective value and the subjective value of the personal level, it is based on intersubjective criteria that are reliably open to scrutiny and do not involve circular reasoning). The value established at the super-personal level through the interpersonal market exchange is ascertained via the very behavior it is employed to explain. Value interpreted at the personal level is necessarily subjective value and not directly available for empirical analysis—at best we rely on self-reports. Values established at the sub-personal level through neurophysiological measurement of dopaminergic action potentials in response to environmental stimuli or neurophysiological measures of emotions that correlate with verbal and other behavioral indices are objective in the sense that they manifest in reliable indices; they provide confirmation or disconfirmation of verbal and other behavioral reports.

This conceptual delineation, concentrating on the explanatory mechanisms that underpin responsive behavior and considered action, differs in several respects from those DPTs that take a more rigid view of the explicatory needs of different styles of consumer choice. First, the intended flexibility allows the appropriate mode of explanation, extensional, or intentional, to be employed to elucidate an observed pattern of consumer choice free of the assumption that it must be *either* stimulus-bound/impulsive *or* governed by executive functions/considered. Second, while some DPTs comprise decision-making units that uncritically span the levels of exposition of their behavioral, cognitive, and neurophysiological components, often switching between them in mid-explanation or using concepts at levels of exposition to which they are not appropriate, the conceptual approach makes explicit the conceptualization in use and the level of exposition at which it is being deployed. Third, it avoids a rigidly bipolar treatment of the automatic and controlled aspects of behavior based on system1/system2 or function1/function2 dichotomies, each of which is supposedly uniquely determinative of a style of activity. Fourth, it reveals the explanatory subtleties inherent in accounting for both responsive behavior and considered action. Our working hypothesis assumed the former to result entirely from impulsivity or automaticity based on momentary unreflecting responses to stimuli and therefore the domain of BPM-E. It assumed also that the latter was exclusively the domain of executive functioning or self-control based on cognitive consideration and reflection and thus the realm of BPM-I. The analysis shows, however, that both kinds of activity require both kinds of explanation in varying measures.

## Data availability statement

The original contributions presented in the study are included in the article/supplementary material, further inquiries can be directed to the corresponding author.

## Author contributions

The author confirms being the sole contributor of this work and has approved it for publication.

## Footnotes

^1^The intentional behaviorist research strategy has been developed in the context of economic psychology of consumer choice. However, the conclusions reached apply generally to human conduct of which consumption is a paradigm, a microcosm of what the behavioral sciences seek to comprehend, and a near-universal facet of experience. Hence, the argument's being posed in terms of *consumer* activity, does not restrict its applicability.

^2^In presenting a basic view of the operant paradigm, it is easy to give the impression that it is a machine-like and unfeeling paradigm for behavioral research. However, I do not wish remotely suggest that operant research is inflexible or mechanistic in its approach to investigation or that it fails to come to terms with alternative conceptual and methodological positions. To the contrary, the radical behaviorist tradition has, especially in recent years, embraced a number of philosophical perspectives that display a catholicity of viewpoints and stimulating intellectual debate. See, e.g., Zilio and Carrara (2021), who draw attention to Staddon's “Theoretical behaviorism,” Rachlin's “teleological behaviorism,” Baum's “molar behaviorism,” Donahoe's “biological behaviorism,” Hayes's “contextual behaviorism,” Ribes-Iñesta's “field-theory behaviorism,” as well as “intentional behaviorism,” which individually and collectively display a vibrant and progressive research programme. Behavior analysis is also home to several stimulating and energetic programmes of “pure” conceptual and empirical research which have transformed traditional behavioral psychology. Prominent examples are the analysis of verbal behavior (e.g., Hayes, 1989), equivalence relations (e.g., Arntzen, 2012; Arntzen and Nartey, 2018, and, in the realm of consumer behavior analysis, Arntzen et al., 2016), and operant behavioral economics (Foxall, 2016c). Many of the insights gained through empirical research in these domains is leading directly into applied behavior analysis which exhibits exciting advances, for example, in the fields of autism, addiction, organizational behavior, psychotherapy and other areas of behavioral change. A prominent example is the adoption by some researchers and practitioners of process-based therapies, e.g., Hayes' (Hayes et al., 2016) Acceptance and Commitment Therapy, ACT, which employ but transcend the operant paradigm to consider previously unexplored mechanisms of action. Another is the study and treatment of autism based on behavior analysis (Leaf et al., 2022). Hence, far from being a lackluster research programme, behavior analysis (to reference only one field of behaviorist endeavor) is alive, cerebrally stimulating and practical.

^3^By “sequences of behavior,” I have in mind a molar pattern of responding that is “behavior patterns extended in time” (as Baum, 2002, 2004 has developed the idea), rather than a concept such as a behavioral chain.

^4^An example of discontinuity is the insensitivity to current stimuli that marks human operant responding in matching tasks when reinforcement schedules are modified (see Foxall and Oliveira-Castro, 2009).

^5^The view of mind pursued here does not presuppose an ethereal ‘mental substance'. The observation that intentional explanation becomes necessary when extensional approaches cannot account for aspects of behavior is a methodological one: it implies no particular ontology (see Ryle, 1949).

^6^Note that my approach differs from the disconfirmation of expectation theories and cognitive dissonance theory. Compare my approach here with, for instance, that of Festinger (1957). I also wish to distinguish my approach from that based on crude intra-personal assumptions such as those found in some cognitive (S-O-R) depictions. For explication, please see Bickel and Yi, 2008.

^7^Berridge and Robinson speak of wanting in two ways: ordinary, cognitive desire, is a matter of the personal level, while ‘wanting' belongs at the sub-personal level, and takes the form of neurophysiological urgings. It may, however, correlate with verbal expressions of desire or craving or by other behavioral indices such as approach. Both imply strong *valuation* of the reinforcer. They ground the conception of value in considerations arising the super-personal level (which is determined by the consumer's learning history), the personal level (which reflects her individual preference structure), and the sub-personal level (which is determined by the physiological urges to which the presentation of the reward or stimuli that predict it gives rise.

^8^Which is always a matter of what is generally understood by liking, i.e., the experience of pleasure, and therefore belongs to the personal level of exposition.

^9^The role of BPM-N is far from confined to the evaluation stage of the intentional behaviorist research strategy. That is, simply the role it has been assigned in the present study (see Foxall, 2024).
